# How is political trust associated with economic and environmental policy prioritization? A longitudinal analysis between 2017 and 2022

**DOI:** 10.1007/s13280-024-02054-z

**Published:** 2024-08-13

**Authors:** Sami Ahonen, Aki Koivula, Jukka Sivonen

**Affiliations:** 1 Department of Social Research, University of Turku, Assistentinkatu 7, 20014 Turku, Finland; 2 INVEST Research Flagship Centre, University of Turku, Assistentinkatu 7, 20014 Turku, Finland; 3 Finnish Institute for Health and Welfare (THL), Mannerheimintie 166, 00271 Helsinki, Finland

**Keywords:** COVID-19, Economic crisis, Environmental policy attitudes, Longitudinal, Political trust, Russian war

## Abstract

In this article, we examine the longitudinal development of economy-over-environment policy priorities in Finland during a period marked by several significant adverse societal events. In addition, we explore the influence of political trust on these priorities over time. We utilize a five-round panel dataset comprising 2155 observations (*N* = 431) to examine both within-individual and between-individual variations from late 2017 to early 2023. Our findings reveal a significant increase in the prioritization of the economy in each observation period following the COVID-19 outbreak. However, this trend is not observed among individuals with high levels of political trust. The within-individual analysis indicates that high political trust moderates, rather than causes, higher economy-over-environment prioritization. Our study highlights the significance of political trust as a preventing factor against the deprioritization of environmental policies, particularly when societies confront significant and intersecting adverse events.

**Supplementary Information:**

The online version contains supplementary material available at 10.1007/s13280-024-02054-z.

## Introduction

The relationship between the economy and the environment has long been a subject of debate. A central issue of this debate is whether it is possible to achieve ecologically sustainable gross domestic product (GDP) growth. This debate includes both optimistic perspectives (e.g., Vazquez-Brust and Sarkis 2012) and more critical viewpoints (e.g., Jackson 2017; Victor 2019). However, there is no evidence supporting the absolute decoupling of GDP growth from environmental harm at the scale, continuity, and speed necessary, for example, to meet the targets of the Paris Agreement (Vadén et al. 2020; Haberl et al. 2020; Vogel and Hickel 2023). It is conceivable that both objectives—GDP growth and a significant reduction in environmental harm—may not be achievable simultaneously (e.g., Vadén et al. 2020). This underscores the importance of prioritizing between economic and environmental goals and highlights the need to study public attitudes regarding this prioritization.

Environmental and climate change are significant public concerns in Europe and Finland (Eurobarometer 2021). Previous research indicates that people prioritize environmental well-being over economic growth, more evidently in countries with higher GDP (Gugushvili 2021). From 1984 to 2022, there was a general inclination among Finns to prioritize environmental protection over their standard of living, although this commitment waned following significant economic shocks, with a heightened focus on economic growth during times of crisis (Metelinen 2023). As Mayer and Smith (2017) demonstrate, environmental concern and willingness to contribute financially to climate protection are primarily influenced by short-term economic conditions; while, long-term economic factors have minimal impact, if any.

In recent years, European countries have faced multiple simultaneous challenges, such as the COVID-19 pandemic, the Russian invasion of Ukraine, and rising inflation (Mišík and Nosko 2023). These events have posed administrative challenges for governments, institutions, and individuals (Homer-Dixon et al. 2022). This period has also intensified economic pressure, raised challenges for environmental policies, and underscored the role of political trust. During the pandemic, nations with higher levels of political trust reported better outcomes, such as reduced mortality rates (Oksanen et al. 2020; Zaki et al. 2022). The influence of political trust on public attitudes has garnered significant scholarly interest, with numerous studies exploring its implications on environmental policy attitudes (Devine 2024, for a comprehensive review). However, studies on economic and environmental prioritization during challenging societal periods have often overlooked the role of political trust in these attitudinal shifts or persistence. This study aims to fill this research gap.

Like many other European countries with a high per capita carbon footprint, Finland needs to reduce its emissions to meet the progressively stricter EU requirement for 2030 and achieve carbon neutrality by 2035, as outlined in the national “Medium-term Climate Change Policy Plan.” This also necessitates public support for mitigation policies. While Finns generally express positive attitudes toward the environment, recent attitudinal shifts (Eurobarometer 2019, 2023; Metelinen 2023), also witnessed in other European countries, have raised concerns about declining support for environmental policies, making Finland an important case to examine. Moreover, recent fluctuations in political trust (Kestilä-Kekkonen et al. 2022) offer an opportunity to examine its connection to environmental prioritization longitudinally.

Using longitudinal panel survey data from the same Finnish respondents between late 2017 and early 2023, we focus on how economy-over-environment policy priorities have evolved during a period marked by multiple adverse societal events. Specifically, we aim to identify how attitude shifts or stability relates to respondents’ political trust. By doing so, we seek to clarify the complex interplay between economic and environmental priorities and shed light on the role of political trust in shaping or solidifying political priorities.

### Changes in environmental policy priorities and recent trends

In Finland (Finnish Government 2023a) and across Europe (Eurobarometer 2019, 2023; Beiser-McGrath 2022), the prioritization of environmental and climate change declined notably between 2019 and 2023, particularly during the first wave of the COVID-19 pandemic and the full-scale Russian invasion of Ukraine. Simultaneously, economic concerns such as inflation, cost of living, and energy have gained significance (Eurobarometer 2023). Although this attitude shift aligns with adverse events and economic turbulence, our primary goal is not to establish causal effects, as this remains challenging even with our longitudinal panel data. However, given the substantial impact of this attitudinal shift on our study, we provide a concise overview of prior research literature explaining similar shifts. A contextual description of the events occurring during our data gathering is provided in the *Materials and methods* section.

In line with the affluence hypothesis (Diekmann and Franzen 1999), economic downturns may decrease environmentalism if the environment is seen as a “superior good” requiring a trade-off in resource allocation. Additionally, these events could impact security as well as economic and political stability, which are seen as drivers of post-materialist values such as environmental concerns (Inglehart 1995). Previous economic shocks have shown a decline in prioritizing environmental protection with rising unemployment rates (Kenny 2019) and perceived economic outlook (Kachi et al. 2015). However, changes in GDP, growth rates (Kenny 2019), or local economic conditions (Mildenberger and Leiserowitz 2017) have not been found to have a significant impact.

Conversely, scholars have explained attitudinal shifts with public agenda theories, focusing on how issues gain prominence in the public sphere. Attitudes may be affected by media agenda-setting (McCombs and Shaw 1972; Lyytimäki et al. 2020) and issue attention cycles (Downs 2016; Sisco et al. 2023) in social and mainstream media, which are key platforms for disseminating political cues that influence environmental prioritization during crises (Mildenberger and Leiserowitz 2017). In addition, the public’s limited worrying capacities (Linville and Fischer 1991) have been used to explain the attention shift toward new emerging issues. However, some studies suggest that crisis concerns may not negate each other (Drews et al. 2022). While these mechanisms likely explain the previously observed shift in public opinion, our aim is not to study the exact causal relationship between crises and attitude changes. Instead, we examine whether a similar shift can be detected in our data, with a particular focus on the moderating role of political trust, which we will discuss next.

### Changing political trust and attitudes

Political trust is a key determinant of environmental policy attitudes, as established by previous research (Konisky et al. 2008; Fairbrother et al. 2021). First, citizens who trust their government are more likely to believe that it will act in their best interests (Brechin and Kempton 1994). Studies indicate that trust in political institutions and leaders increases endorsement and compliance with environmental policies (Fairbrother et al. 2021). Conversely, low trust in the government can lead to skepticism toward environmental policies, perceiving them as being influenced by special interest groups or as a means for the government to exert greater control over citizens. Consequently, a lack of political trust has been observed to correlate with decreased support for environmental policies (Fairbrother 2016).

The importance of trust lies in its ability to reduce the complexities of social life by establishing a foundation of confidence in the reciprocal actions of others (Luhmann 1979). Trust refers to the confidence of a subject (A) that an object (B), which can be a person, group, or institution, will perform as expected in a given situation (X) (PytlikZillig and Kimbrough 2016). Enhancing cooperation relies on trust, as it enables the maintenance of social order without excessive legislation or regulation (Macy and Skvoretz 1998; Balliet and van Lange 2013). Trust is closely linked to the concepts of risk and uncertainty. Without uncertainty associated with the actions of the object, the subject would not need to have confidence that their expectations would be met (Luhmann 1979; Schilke et al. 2021). Given that trust is related to unknown outcomes, it reduces the need for constant monitoring, thereby avoiding excessive legislation and regulation (Robbins 2016).

Political trust can be understood as generalized and diffused trust in key institutions, where the object of trust is not a particular political actor but rather the system and its actors (Citrin and Stoker 2018). In this context, political trust emerges as an essential factor in maintaining the functioning and sustainability of any democratic society, enabling citizens to have faith in their government and its ability to address their concerns and represent their interests (Easton 1975; Hetherington 1998).

Given that political trust operates reciprocally, a climate of trust allows policymakers to issue laws, instructions, and guidelines with the confidence that citizens will comply (Hooghe and Marien 2013; Lalot et al. 2022). Conversely, low levels of political trust have been linked to feelings of alienation, cynicism, and disengagement from politics (Fox 2021). Similarly, political distrust may undermine support for collective solutions, as perceptions of the administration’s incapability or corruption may hinder monitoring and cooperation (Linde 2018). As a result, individuals may opt out of cooperation, doubting that others will cooperate as well (Jagers et al. 2020).

In the realm of politics, political trust is crucial for gaining public support for policies that entail inherent risk. Such policies involve a certain level of uncertainty regarding their potential beneficial or unfavorable outcomes (Rudolph 2017). Environmental risks are typically seen as “wicked problems” (Ludwig 2001), characterized by contingency, uncertainty, and a lengthy time frame; by contrast, economic risks, such as unemployment, economic growth, and inflation, are perceived as more immediate and focused on shorter-term gains or losses (Levin et al. 2012).

Moreover, environmental policy often necessitates collaborative efforts across multiple jurisdictions and involves a diverse set of stakeholders; whereas, economic policy decisions are typically driven by national interests and objectives (Schneider and Volkert 1999). Environmental protection usually addresses longer-term issues; whereas, economic policies often focus on shorter-term outlooks and problem solving. Consequently, political trust plays a distinct role in shaping attitudes toward economic policy compared to environmental policy.

Several studies have indicated that political trust shapes attitudes by serving as a heuristic (Hetherington and Globetti 2002). The trust-as-heuristic theory emphasizes that trust functions as a cognitive shortcut, especially in situations of uncertainty when individuals lack the capacity to process and evaluate information on their own (Rudolph 2017). As a result, individuals may express support or opposition toward political governance based on their level of political trust. Furthermore, political trust not only shapes opinions and attitudes but also an individual’s willingness to make material and ideological sacrifices (Hetherington 2005). In this manner, the heuristic dimension of political trust overlaps with its role in reducing complexity. Political trust empowers individuals to take risks and make sacrifices, assured that government decisions will ultimately benefit them (Jacobs and Matthews 2017; Fairbrother et al. 2021). Recent experimental studies suggest that political trust does not necessarily influence people’s policy preferences without an element of uncertainty; however, it can be neutral if people are provided with more information about policy effects and choices (Christensen and Rapeli 2021; Devine et al. 2023).

Political trust, once established, is relatively stable at the individual level despite momentary changes (Devine and Valgarðsson 2024). However, during times of external crisis, there is often a notable increase in citizens’ trust in politicians and national administrations, a phenomenon often described as the “rally ‘round the flag” effect (e.g., Baum 2002). Several explanations for this effect exist, such as the emotional response to fear, which can lead individuals to trust authorities (Hetherington and Nelson 2003). In times of crisis, policymakers can also become opinion leaders with central access to first-hand information; thus, their decisions or views may not be subject to criticism, even in the media (Baker and Oneal 2001). This perspective also resonates with the trust-as-heuristic theory, which highlights how political trust effects on environmental policy support are activated, particularly in situations of high uncertainty whereby citizens have little information (Fairbrother 2019; Devine et al. 2023). Therefore, the role of political trust in shaping attitudes may be especially important during crises, when political trust is strengthened and uncertainty is increased. Conversely, crisis-related fluctuations in political trust may not directly affect other policy preferences that are less salient during crises (Hetherington and Husser 2012).

However, not all crises increase confidence in political institutions. Previous studies have shown that economic crises, in particular, increase dissatisfaction with governance, which, in turn, is reflected in declining political trust (van der Meer 2017). The hardships faced by individuals during economic downturns, such as job losses, financial instability, and increased cost of living, may lead to frustrations and disappointments with public authorities (van Erkel and van der Meer 2016). The perception that political leaders are unable to respond effectively to economic challenges may further erode political trust. The negative effects of economic crises on political trust extend beyond immediate economic concerns and spill over into the other domains of politics, such as the environment. During economic struggles, people’s attention and priorities may shift to more immediate economic concerns, diminishing their focus on environmental issues, especially if they lack trust in the ability of political leaders to navigate the crisis. By contrast, political trust can provide resilience in the face of uncertainty and the capacity to make short-term economic sacrifices for long-term policies (Jacobs and Matthews 2017). Thus, political trust may allow individuals to sacrifice short-term economic benefits (their own or the state’s) for longer-term environmental benefits, thereby stabilizing their attitudes toward environmental policies during economic shocks.

### This study

#### Hypotheses

Our study investigates the shifting prioritization of economic over environmental politics in Finland within a fluctuating economic context, exploring the role of political trust and its potential moderating effect on these attitudes over time. First, our analysis assesses the development of the economy-over-environment prioritization across five survey rounds from late 2017 to late 2022. Drawing on extensive polls and surveys conducted in recent studies and available literature on the impact of crises on political prioritization (Eurobarometer 2023; Finnish Government 2023a; Drews et al. 2022; Gregersen et al. 2022), we expect that public prioritization has predominantly shifted from environmental to economic concerns. Based on this, we propose the following hypothesis:

##### H1

The prioritization of economic policy over environmental policy has increased within our sample after the onset of the COVID-19 pandemic.

Subsequently, we examine the role of political trust in explaining attitude fluctuations during a crisis, building upon previous research presenting political trust as a significant determinant of environmental policy attitudes (Konisky et al. 2008; Fairbrother et al. 2021). It has been noted that individuals with high levels of political trust are more likely to believe in reciprocal efforts and collective action when addressing long-term environmental crises (Jagers et al. 2020). This effect of political trust may be especially significant during crises, as the urgency of short-term issues can distract many people from focusing on longer-term problem solving. We anticipate this pattern in how political trust predicts attitudes, both cross sectionally and longitudinally. With this in mind, we propose the following hypotheses:

##### H2

Individuals with greater trust in political institutions prioritize environmental policy relative to economic policy more than individuals with low political trust.

##### H3

As the political trust of respondents increases during the crisis period, their prioritization of economic over environmental policy decreases.

Second, we draw on the trust-as-a-heuristic thesis, which states that political trust influences people’s attitudes, particularly in high-risk situations involving informational uncertainty and short-term financial sacrifices (Rudolph 2017; Fairbrother et al. 2021). Consequently, individuals with high political trust may be less susceptible to the impact of adverse societal events. We propose that individuals with high political trust are less likely to alter their attitudes amidst the crises:

##### H4

Higher political trust reduces the likelihood of increasing the prioritization of economic policies over environmental policies during periods of societal uncertainty.

## Materials and methods

### Participants

Our data come from the “Digital Age in Finland” survey, which tracks the same group of participants from 2017 to 2023 across five measurement points. The first survey was conducted in December 2017; while, the last one took place from December 2022 to January 2023. The initial data (T1) were collected from two samples of Finns aged 18–74, with a total of 3724 participants and a response rate of 30.8%. The second phase (T2) followed in spring 2019, with 1134 responses (30.5% of T1 respondents). The third phase (T3) was conducted in May–June 2020 during the first wave of the COVID-19 pandemic, with 735 respondents (64.8% of T2 respondents). The fourth phase (T4) was administered in December 2021, with 543 respondents (73.9% of T3 respondents). The final phase (T5) was implemented in December 2022, with 431 respondents (79.4% of T4 respondents).

Only those who participated in all five measurement points were included in the study. The data were quite representative in terms of age and sex but skewed toward highly educated people. Attrition was a significant issue, with many participants discontinuing their engagement across survey rounds, which added variability to the data’s representativeness. We have detailed this in Appendix S1 (Table S1), focusing on gender, age, education, and party affiliation distributions at different measurement points. In the analyses, we accounted for the data’s skewness and attrition by using post-stratification weights calibrated on age, gender, and education (detailed in Appendix S2, Table S2). We first calculated the weights by age and gender, then further adjusted them by educational level (Deville and Särndal 1992).

All participants consented to the collection and research use of their identifying information and sensitive COVID-19-related issues. They were also asked for follow-up survey participation consent and permission to store contact information for outreach.

### Study context

The study context is detailed in Appendix S3 (Table S3; Figure S1). During the first observation period (T1), 14% of Finns considered the environment, climate, and energy among the top two most important policy issues (Eurobarometer 2017). This percentage surged to 35% around T2 (Eurobarometer 2019) during extensive discussions on climate change before the forthcoming parliamentary elections, often referred to as “the climate election.” By the time of T3, during the first wave of COVID-19, the percentage had decreased to 24%. Notably, before T3, Eurobarometer permanently altered the wording by omitting the term “energy” (Eurobarometer 2020). Media attention to climate change also clearly decreased around this time but slowly rebounded in the following months (Lyytimäki et al. 2020). At T4, prioritization was at 22%, and by T5, it had further decreased to 15% (Eurobarometer 2022; 2023).

In terms of concern for other societal adverse events, pandemic-related concern was relatively high at T3 and T4 but decreased significantly at T5, the only observation period after the Russian full-scale invasion of Ukraine. Ten months into the invasion at T5, 60% of Finns expressed concern about the war’s impact on the Finnish economy (Finnish Government 2023b).

Economic conditions also shifted during this period. Inflation remained relatively moderate from T1 to T3, surged before T4, and spiked notably at T5 (Statistics Finland 2024a). Around T3, the national economy shrank due to COVID-19 restrictions but began to rebound in early 2021, except for disruptions during the new COVID-19 wave at the time of T4 (Honkatukia 2022). Consumer confidence fluctuated near the average at T1 and T2, declined to relatively low levels at T3 with the onset of the pandemic, rebounded to near-average levels at T4 but fell again to an all-time low at T5 amid the war-induced energy crisis (Statistics Finland 2024b).

Politically, the right-leaning Sipilä administration was in power during T1 and T2; while, the more left-leaning government led by Social Democrat Sanna Marin was in office from T3 to T5. This latter government included the Green Party, the red-green Left Alliance, the agrarian-centrist Centre Party, and the liberal Swedish People’s Party of Finland and showed significantly more ambition in environmental politics than the former. Nevertheless, we do not believe this significantly impacted the shift in environmental prioritization, as a very similar shift was observed across Europe during the same timeframe (Eurobarometer 2019; 2023). Political trust, measured at a more general level, does not account for the current government. However, our statistical analysis accounts for the political party the respondents voted for and their pre-election political trust in the 2019 elections.

### Measures

#### Economic policy over environmental policy

Table 1 presents the variables analyzed in this study along with their descriptive statistics. Our outcome variable measures the prioritization of economic policy over environmental policy. Participants were asked, “How do you relate to the following: Placing economic policy ahead of environmental policy.” Responses were recorded on an 11-point scale (0 = “very negatively,” 10 = “very positively”), treated as a continuous variable.

#### Political trust

We assess political trust in representative institutions, including the parliamentary system and key actors. Participants rated the trustworthiness of “the Finnish parliament,” “political parties,” and “politicians” on a five-point scale (1 “not trustworthy at all”–5 “very trustworthy”). Subsequently, we aggregate these ratings into a single-sum variable (Marien 2017), with a Cronbach’s alpha of 0.87, indicating good consistency. This measure of political trust is treated as a time-variant continuous variable, allowing for analysis of changes in trust levels throughout the study.

#### Control variables

We incorporate several socio-demographic and attitudinal predictors in our models based on prior research that indicates their influence on policy attitudes. These include age, education, gender, financial situation, political orientation, and party preference. Age is measured in years; while, gender is treated as a binary variable. Education is classified by whether participants have achieved at least a “tertiary” education level. Financial situation is rated on a scale from 0 to 10 (0 “very bad,” 10 “very good”).

To determine party preference, participants were asked about their voting choices in the 2019 parliamentary elections. During the analysis, we compare respondents based on whether their preferred party was in the government during rounds 3 to 5 using a time-invariant dummy variable. Political orientation is assessed by participants’ self-placement on a left–right scale (0 “extremely left,” 10 “extremely right”). Education, financial situation, and political orientation are treated as time-varying variables (level 1); while, the other control variables are considered as time-invariant, only varying between individuals (level 2).

#### Analysis procedure

We employ a longitudinal research design to examine the relationship between changing economic-over-environmental policy priorities and political trust over time. Our analysis utilizes the complete dataset and employs random effects within-between (REWB) models (Bell et al. 2019) with random intercept and cluster-robust standard errors to account for the correlation between observations among individuals in the panel data. The REWB models combine fixed and random effect approaches, allowing us to analyze both within-person changes and between-person differences over time while controlling for relevant covariates. The REWB models calculate the within effects by measuring the deviation from the respondent-specific mean and the between effects by incorporating the respondent-specific mean. At the within-level, we analyze only the observed changes in the independent variable, such as political trust, between time points, while at the between-level, we consider all possible observations. This method allows us to analyze how a within-level change in the independent variable predicts changes across time points in the dependent variable while simultaneously analyzing how the respondent-specific mean in the independent variable predicts the difference between respondents in the dependent variable.

First, we examine the temporal changes in the economy-over-environment policy prioritization within individuals. In the second model, we assess the impact of political trust within and between individuals. The third model includes both time and political trust. The interaction between time and political trust is added to the fourth model, and the relevant covariate variables are added to the fifth model. The sixth model includes all variables used at different levels. The model incorporates a random intercept; while, the slope is typically fixed at the individual level. Additionally, we conduct robustness checks by considering each component of political trust separately and by adding the random slope model of political trust to account for random variations in the relationship between political trust and the dependent variable at the individual level (Bell et al. 2019). We perform the REWB analysis with Stata 17 using the mixed command and use the coefplot command to illustrate the main results in figures.

**Table 1:** Descriptive statistics of applied variables. Measurement time in parentheses

Variable	Obs	Mean	Std. Dev	Min	Max
Economy versus Environment (T1)	431	3.633	2.506	0	10
Economy versus Environment (T2)	431	3.543	2.660	0	10
Economy versus Environment (T3)	431	3.896	2.690	0	10
Economy versus Environment (T4)	430	4.142	2.836	0	10
Economy versus Environment (T5)	430	4.379	2.889	0	10
Political trust (T1)	431	2.526	0.838	1	4.333
Political trust (T2)	431	2.488	0.807	1	4.333
Political trust (T3)	431	2.785	0.821	1	5
Political trust (T4)	430	2.720	0.826	1	5
Political trust (T5)	430	2.725	0.832	1	5
Gender (T1)	431	0.457	0.499	0	1
Birth cohort (T1)	431	1968.52	15.404	1943	1999
Party voted (T2)	428	0.530	0.500	0	1
Higher education (T3)	430	0.512	0.500	0	1
Financial situation (T3)	431	6.480	2.392	0	10
Political orientation (T3)	427	4.768	2.572	0	10

## Results

We proceed by analyzing the changes in the dependent variable over time in relation to political trust during the data gathering period. Table 2 presents the coefficients from REWB models M1–M6. In the models, the intraclass correlations (ICC) indicate that approximately 60–72% of the overall variation in the outcome variable is attributed to level 2 factors. Variance analyses at levels 1 and 2 suggest greater variability between individuals than within the same individual over time. Controlling for background variables reduces this disparity, suggesting that individual variability may be explained by differences in these background factors, underscoring the relative consistency in attitudes. This highlights the importance of considering individual characteristics and contextual factors when interpreting the outcomes and suggests that the priorities of specific individuals exhibit relatively minor variations compared to the differences between individuals.

**Table 2:** Predicting economy-over-environment policy preferences according to political trust and time. Coefficients from the weighted REWB models. Clustered standard errors in parentheses, ***p < 0.001, **p < 0.01, *p < 0.05

	M1		M2		M3		M4		M5		M6	
	B	se	B	se	B	se	B	se	B	se	B	se
*Time effect (ref. T2)*												
T1	0.089	(− 0.112)			0.084	(− 0.113)	0.351	(− 0.403)	0.106	(− 0.118)	0.271	(− 0.413)
T3	0.368**	(− 0.116)			0.332**	(− 0.128)	0.609	(− 0.422)	0.392**	(− 0.128)	0.462	(− 0.43)
T4	0.789***	(− 0.146)			0.761***	(− 0.152)	2.112***	(− 0.639)	0.799***	(− 0.15)	2.002**	(− 0.644)
T5	1.049***	(− 0.15)			1.020***	(− 0.155)	2.536***	(− 0.616)	1.024***	(− 0.151)	2.423***	(− 0.614)
Political trust (within)			0.335**	(− 0.117)	0.109	(− 0.115)	0.165	(− 0.247)	0.057	(− 0.124)	0.11	(− 0.243)
Political trust (between)			− 0.379	(− 0.216)	− 0.382	(− 0.217)	− 0.129	(− 0.255)	− 0.251	(− 0.18)	− 0.037	(− 0.227)
*Time interaction*												
T1 * political trust (within)							0.529	(− 0.37)			0.273	(− 0.377)
T3 * political trust (within)							− 0.469	(− 0.394)			− 0.228	(− 0.391)
T4 * political trust (within)							− 0.139	(− 0.34)			− 0.022	(− 0.349)
T5 * political trust (within)							− 0.265	(− 0.394)			− 0.227	(− 0.404)
T1 * political trust (between)							− 0.078	(− 0.155)			− 0.05	(− 0.163)
T3 * political trust (between)							− 0.087	(− 0.147)			− 0.023	(− 0.149)
T4 * political trust (between)							− 0.523*	(− 0.217)			− 0.473*	(− 0.22)
T5 * political trust (between)							− 0.582**	(− 0.212)			− 0.541*	(− 0.214)
*Controls*												
Voted for govt party in 2019									− 0.12	(− 0.278)	− 0.118	(− 0.279)
Left–right (within)									0.098*	(− 0.042)	0.090*	(− 0.04)
Left–right (between)									0.537***	(− 0.057)	0.533***	(− 0.057)
Subj. economic situation (within)									0.088	(− 0.051)	0.08	(− 0.048)
Subj. economic situation (between)								0.105	(− 0.064)	0.106	(− 0.064)	
University or UAS (within)									− 0.026	(− 0.199)	0.083	(− 0.188)
University or UAS (between)									− 0.648**	(− 0.223)	− 0.648**	(− 0.224)
Birth year									− 0.018*	(− 0.008)	− 0.018*	(− 0.008)
Female									− 0.437	(− 0.228)	− 0.434	(− 0.229)
Constant	3.318***	(− 0.174)	4.756***	(− 0.58)	4.326***	(− 0.589)	3.681***	(− 0.686)	36.908*	(− 15.175)	36.415*	(− 15.306)
Variance at level 1 (within individual)	2.084	(0.180)	2.265	(0.212)	2.082	(0.180)	2.023	(0.167)	2.054	(0.182)	2.006	(0.171)
Variance at level 2 (between individual)	5.419	(0.441)	5.284	(0.443)	5.333	(0.442)	5.339	(0.442)	3.137	(0.297)	3.170	(0.301)
ICC at the ID level	0.722	(0.028)	0.700	(0.031)	0.719	(0.028)	0.725	(0.027)	0.604	(0.034)	0.612	(− 0.0334)
Observations	2138		2137		2137		2137		2091		2091	
Number of individuals	428		428		428		428		425		425	

Next, we examine the time trend and investigate the predictive impact of political trust on both individual differences (between effect) and changes over time (within effect).

**Fig. 1:**
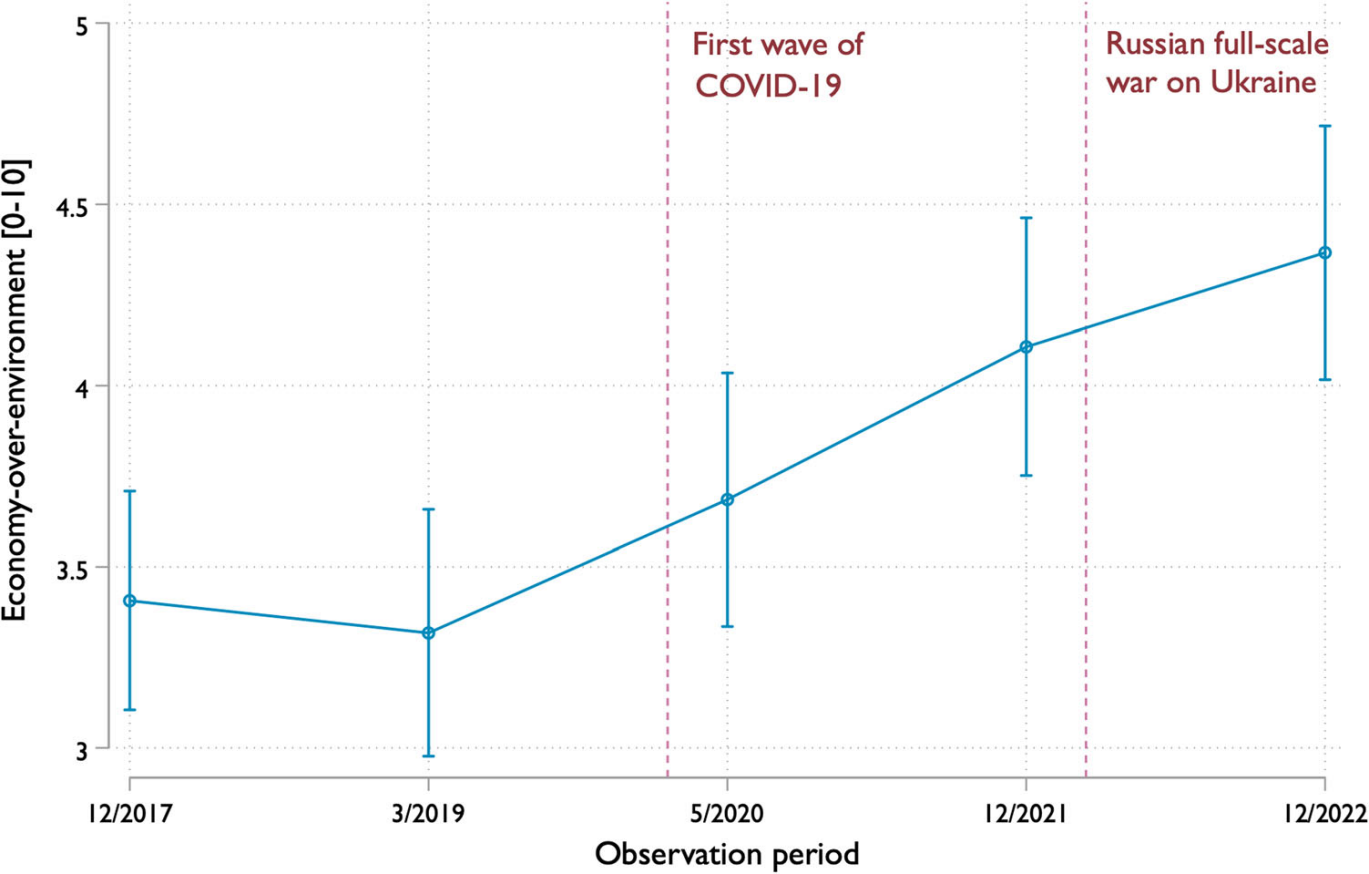
Economy-over-environment prioritization 2017–2023. The predicted scores from the weighted linear model of economy-over-environment prioritization on observation period with random intercepts by ID. Estimates with 95% confidence intervals

In line with H1, although there was no difference between T1 and T2 in M1, a trend toward higher prioritization of economic over environmental policies emerged in each subsequent round compared to the last pre-COVID-19 round, T2. Figure 1 illustrates this trend. To mitigate potential confounding variables, such as political orientation, party support, and socioeconomic factors, we introduce control variables separately alongside the time effect in M5. However, the effect at T3 becomes statistically insignificant when considering the time-trust interaction in M4 and M6, suggesting that the shift in prioritization did not immediately follow the onset of COVID-19 on T3. We will revisit this finding in the discussion.

Regarding political trust, the effect between individuals is negative and nearly significant (*p* = 0.078) in M3. This suggests that individuals reporting high political trust prioritize environmental policy more than those with low levels of trust, consistent with prior research (e.g., Konisky et al. 2008; Fairbrother et al. 2021). The effect size increases after controlling for covariates in M5; however, the statistical significance diminishes substantially (*p* = 0.166). Given the potential influence of the small sample size on the reported *p*-value, our models provide partial support for H2.

Considering the within effect of political trust, M2 is the only model to demonstrate a significant effect, suggesting that an increase in an individual political trust (within effect) leads to a greater prioritization of economic policy. This finding contradicts H3 and could be attributed to the concurrent rise in political trust following the onset of COVID-19 between rounds 2 and 3 (Kestilä-Kekkonen et al. 2022). Once the time effect and other variables are considered alongside political trust in M3 and M5, the within effect of political trust loses significance; while, the time effect for T4 and T5 remains statistically significant, implying that the positive correlation in M2 is likely due to the rally effect. In conclusion, our analysis does not support H3, as a decrease in political trust does not correspond to a higher prioritization of economic policy.

In M4, we control for time-trust interactions to evaluate the variability of the political trust effect across various time points. The interaction is significant for the between effect of political trust at both T4 and T5, suggesting that the predictive role of political trust on prioritization differed between these time points and T2. Introducing control variables in M6 to accurately assess time-trust interaction effects does not significantly alter the results. This finding supports H4.

**Fig. 2:**
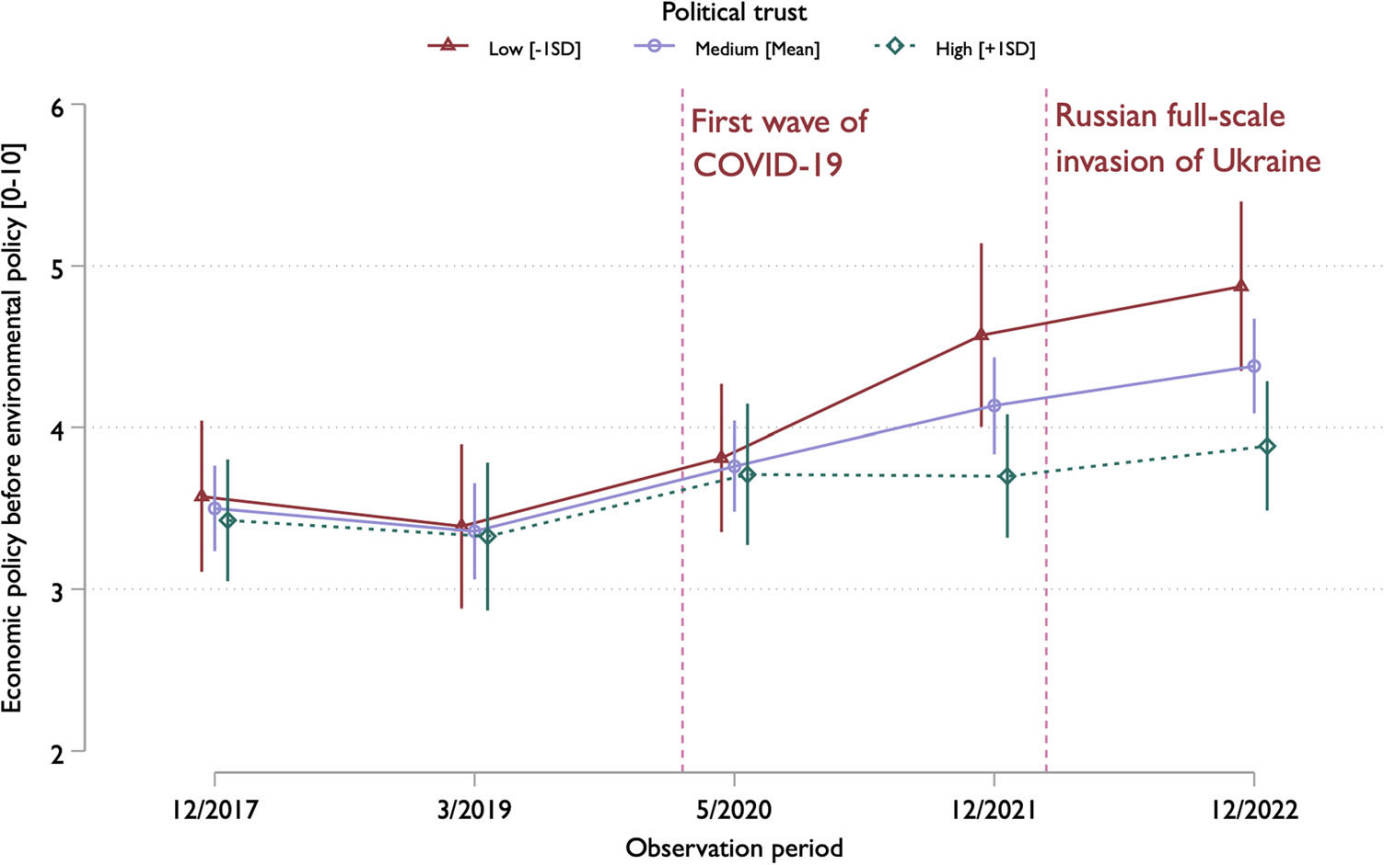
Economy-over-environment policy preferences by political trust and round 2017–2023. The predicted scores from the weighted REWB model (M6). Estimates with 95% confidence intervals

However, achieving statistical significance alone does not sufficiently capture the nuances of the interaction. Thus, we calculate the M6 marginal effects using STATA’s margins commands to plot the results (Fig. 2). This illustrates how low political trust amplifies the economy-over-environment prioritization over time while high political trust stabilizes it. The difference between high and low political trust is not noticeable before the pandemic nor in the first post-pandemic round at T3, as noted earlier. Nevertheless, it becomes apparent in T4 and is even more pronounced in T5, indicating that the moderating effect of political trust is tied to specific periods in our data.

Regarding the control variables, in M5–M6, only lower education level, older age (between individuals), and right-wing political orientation (both within and between individuals) statistically significantly increase the prioritization of the economy. Voting for a government party in the 2019 election, subjective economic situation, and gender do not have a statistically significant effect on the dependent variable.

To conclude, the only statistically significant main effect of political trust on the dependent variable is the within-individuals effect, and it is associated with a specific time point. The between effect was nearly significant in M3, possibly owing to our limited sample size. In addition, the significant interaction effects of between-level political trust and time in M4 and M6 manifest the role of political trust in shaping attitudinal changes over time, supporting H4.

Consistently similar results were obtained in a robustness check where each component of the political trust sum variable—trust in parliament, politicians, and political parties—was analyzed separately (Appendix S4, Figures S2–S4). Similarly, a random slope model accounting for the random variation in the correlation between political trust and the dependent variable at the individual level yielded similar results. The interaction term of political trust (between individuals) and rounds T4 and T5 remained significant.

Although the Finnish government is not included in our political trust variable components, we conducted a robustness check to determine if the results were influenced by supporters of the post-election government being inherently more environmentally conscious. Two distinct interaction models substituting between-political trust with political trust at T1 and political trust at T2 yielded very similar results (Appendix S4, Figures S5–S6), suggesting that the variation is not due to changes in government.

## Discussion

This study examined the evolution of economy-over-environment policy prioritizing and the moderating role of political trust among Finnish respondents during a period overshadowed by several highly salient societal challenges, including the COVID-19 outbreak, the Russian full-scale invasion of Ukraine, and economic turbulence. Our findings contribute to the existing literature by highlighting how political trust longitudinally influences the balance between environmental and economic priorities during challenging periods.

Consistent with H1, our longitudinal five-round survey from late 2017 to late 2022 reveals a significant increase in aggregate economy-over-environment policy prioritization among respondents. This trend coincided with multiple adverse societal events in Europe and deteriorating economic conditions. Similar shifts have been observed in other countries following the COVID-19 outbreak (Beiser-McGrath 2022; Drews et al. 2022; Gregersen et al. 2022). However, our study extends this understanding by examining the impact after the onset of the Russian invasion of Ukraine. In our data, the prioritization of economic policy peaked following the Russian full-scale invasion of Ukraine (Finnish Government 2023b), coinciding with mounting economic pressures (Statistics Finland 2024a). However, establishing causality necessitates further investigation, as our current data do not pinpoint specific contextual causal factors. Furthermore, this study is the first to demonstrate the occurrence of this phenomenon within our follow-up group in Finland. The results also indicate that the variance between individuals is greater than that observed for the same individual over time. This finding contributes to the literature on environmental attitudes by indicating a certain consistency in these preferences over time.

The phases of the crisis must also be taken into account, particularly the increase in economic uncertainty as the crisis persists. As depicted in Fig. 2 and observed in the third phase (T3), divergence in attitudes among respondents with differing levels of political trust did not immediately emerge following the onset of the COVID-19 pandemic; rather, it became more pronounced in subsequent waves. This phenomenon suggests that shifts in economy-over-environment prioritization evolve gradually rather than abruptly at the outset of societal adversity. Previous analysis has similarly noted a delay in the impact of economic uncertainty on environmental attitudes in Finland (Metelinen 2023). Moreover, it is plausible that while the prioritization of economic policy increased during the crisis period, the prioritization of environmental policy may have remained relatively stable if assessed independently.

Societal crises may intensify pressures on post-materialist values, such as economic affluence, security, and political stability (Inglehart 1995), potentially influencing the shift toward materialistic priorities. While our data do not allow precise conclusions about the impact of each crisis on attitude shifts, the results align with expectations of long-term effects. In addition, while our results only consider a non-representative sample in Finland, other studies using a similar dependent variable have found resembling evidence in other countries during the Great Recession (Kenny 2019) and the COVID-19 pandemic (Beiser-McGrath 2022).

Regarding political trust, H2 received only partial support, as the positive correlation between political trust and environmental policy prioritization among individuals was nearly statistically significant. However, a closer investigation revealed that the between effect of political trust is more clearly tied to specific periods in our data: Individuals with high political trust reported stable attitudes over time, whereas those with low political trust increasingly prioritized the economy over the environment, supporting H4. Nevertheless, H3 lacked support, as an increase in political trust over time did not result in changes in economy-over-environment policy preferences within individuals after adjusting for the time period.

While this finding emphasizes the importance of political trust in the environmental policy context, consistent with previous studies (Konisky et al. 2008; Fairbrother et al. 2019, 2021), it also highlights a crucial observation: fostering political trust at an individual level does not immediately influence environmental policy preferences. This result is consistent with recent findings that political trust universally translates into policy preferences in all circumstances (Christensen and Rapeli 2021; Devine et al. 2023). In light of our findings, it appears that the notable increase in political trust following the COVID-19 outbreak did not lead to enhanced prioritization of environmental policy at the individual level.

Our results confirmed the fourth hypothesis that political trust may safeguard public attitudes from shifting toward economy-over-environment policy prioritization during challenging periods. This importance of political trust was highlighted at the fourth and fifth measurement points (i.e., at the end of 2021 and toward the end of 2022), which are periods marked by significant economic uncertainties in Finland. In this regard, our findings align with the notion that political trust acts as a heuristic factor, especially during uncertain times and when material sacrifices are at stake (Jacobs and Matthews 2017; Rudolph 2017).

Trust in political institutions is especially crucial for environmental policy due to the central role of legislative institutions in addressing large-scale and long-term collective problems. High political trust was associated with consistent attitudes over time, possibly stemming from confidence in political institutions’ capability to manage concurrent and complex short-term crises. As a result, economic risks may be perceived as less urgent and severe, allowing attention to focus on longer-term issues such as environmental degradation and climate change (Fairbrother et al. 2021). Overall, our findings on the impact of political trust are consistent with the understanding that it tends to persist over time (Devine and Valgarðsson 2024). While there may be temporary fluctuations in political trust, these generally do not alter people’s fundamental preferences or attitudes. Instead, basic levels of political trust appear crucial in shaping individuals’ attitudes toward different policies during crises.

In terms of policy implications, the multifaceted nature of multiple societal crises demands a coordinated response that balances short-term economic concerns with long-term crisis management (Hukkinen et al. 2022). This trade-off has been salient in recent years, with differing opinions on supporting low-cost energy for economic relief (especially in the industry and fossil energy sector) versus viewing crises as opportunities for sustainable transition (Mišík and Nosko 2023). Without widespread political trust, politicians may opt for short-term solutions to mitigate immediate negative consequences (Artinger et al. 2019), especially during challenging times. Nonetheless, our findings indicate that political trust, rather than being a causal factor, primarily serves to stabilize attitudes toward environmental policy during crises. This suggests that while fostering political trust may not directly increase the prioritization of environmental policies, it can enhance resilience in tackling enduring environmental challenges.

Considering the timing of our study, it is crucial to acknowledge the potential influence of the COVID-19 period on the observed results. Previous research has indicated an increase in trust in political institutions during the initial stages of the outbreak (Kestilä-Kekkonen et al. 2022). Thus, the relationship between political trust and environmentalism may have been confounded by other variables associated with the pandemic (Kritzinger et al. 2021; van der Meer et al. 2023). Future research should consider the timing and circumstances of data collection, as well as potential mediators and moderators that could influence the association between political trust and policy preferences.

Despite the strengths of our longitudinal study, we must acknowledge its limitations. First, although we used post-stratification, the generalizability of our findings should be treated with caution due to possible differences in respondents’ demographic characteristics, especially education, and the risk of self-selection bias in panel data. Second, the small sample size limits the generalizability and reliability of our findings, particularly in moderation analyses.

It is also worth noting that the Finnish government and its parties changed in 2019 (between T3 and T4), which may have affected the associations between political trust and economy-over-environment prioritization. To control for this effect, as noted in the Results section, we tested substituting respondents’ political trust to all phases from the first two phases, which did not significantly affect the results. To validate and extend our conclusions, future studies using larger and more diverse longitudinal samples are needed to gain a more robust understanding of the temporal dynamics between political trust and prioritization of environmental policies. The interplay between time and political trust might differ according to certain features, such as political orientation or perceiving the crises as interconnected (Drews et al. 2022). Future research could refine hypotheses and employ more comprehensive data for a more precise investigation. In addition, experimental research is needed to directly measure the impacts of crises. Future studies should also conduct between-country comparisons, considering the variability of different political situations, including those related to climate change. Assessing these factors would offer valuable insights into the mechanisms that influence changes in environmental attitudes.

Lastly, our dataset prevents us from ascertaining whether the prioritization of environmental policy will return to previous levels in the future. Other descriptive findings indicate that the impact of economic shocks on environmental concern might be temporary (Hartmann and Pfeisendörfer, 2024). However, the willingness to compromise living standards for environmental protection, which suggests an economic trade-off, has declined in Finland since the late 1980s, particularly during significant economic fluctuations, with incomplete recovery observed after each shock (Metelinen 2023). Future studies should delve deeper into the long-term repercussions of societal adversities and how they relate to attitudes related to the economy and other societal concerns.

## Supplementary Information

Below is the link to the electronic supplementary material.

Supplementary file1 (PDF 903 KB)
