# Supplementary material for: How is political trust associated with economic and environmental policy prioritization? A longitudinal analysis between 2017 and 2022
Source: Ambio. 2024 Aug 13;54(1):43–56. doi: 10.1007/s13280-024-02054-z (PMC11607284; doi:10.1007/s13280-024-02054-z)

Ambio

Supplementary Information
This supplementary information has not been peer reviewed.

Title: **How is political trust associated with economic and environmental policy prioritization? A longitudinal analysis between 2017–2022**

The authors have no conflicts of interest to declare. All co-authors have seen and agree with the contents of the manuscript and there is no financial interest to report. We certify that the submission is original work and is not under review at any other publication.

Appendix S1. Data representativeness and attrition

Table S1 shows the representativeness of data in relation to population figures in terms of gender. age. education and party affiliation.

Across the survey rounds. we observe some noteworthy shifts. The gender distribution exhibits a slight disparity. with more men (54%) than women in the most recent measurement. The age structure representation has undergone a gradual normalization. as the initial overrepresentation of respondents born in the 1950s has diminished over time. However. the most significant transformation in the data's representativeness is seen in terms of education. There has been a progressive increase in the proportion of individuals with higher education degree (Master’s or Bachelor’s level). indicating that individuals with lower educational backgrounds are more likely to have dropped out of the survey earlier than their highly-educated counterparts. Conversely. the distribution of respondents affiliated with different political parties has remained relatively stable from one round to the next.

It's worth noting that the influence of attrition is relatively modest in this study. Given our primary focus on tracking changes within individuals over time. Existing research has suggested that attrition tends to impact comparisons between different respondents and direct estimations of means and proportions. Nevertheless. it does not substantially affect the relationships between variables over time (Gustavson et al., 2012).

Table S1. Representativeness of the data in relation to the population in different rounds

	t1		t2		t3		t4		t5		Population*
	12/2017		3/2019		5/2020		12/2021		12/2022		
	N	%	N	%	N	%	N	%	N	%	%
Gender											
Male	1852	49.91	567	50.18	383	52.18	288	52.94	236	54.00	50.2
Female	1854	49.96	559	49.47	345	47.00	252	46.32	196	44.85	49.8
Year of birth											
1990-1999	392	10.57	141	12.47	91	12.38	70	12.96	50	11.74	16.6
1980-1989	497	13.4	196	17.33	133	18.1	94	17.41	91	21.36	17.6
1970-1979	476	12.83	181	16	128	17.41	101	18.70	79	18.54	16.3
1960-1969	691	18.63	188	16.62	136	18.5	101	18.70	80	18.78	17.9
1950-1959	965	26.02	248	21.93	147	20	109	20.19	77	18.08	17.7
1943-1949	688	18.55	177	15.65	100	13.61	65	12.04	49	11.50	13.9
Education level											
Primary	456	12.56	64	5.76	34	4.72	20	3.73	18	4.21	18.0
Secondary	1931	53.17	550	49.5	338	46.94	240	44.78	184	42.99	46.1
Higher	1245	34.28	497	44.73	348	48.33	276	51.49	226	52.80	35.8
Party choice before the parliamentary election 2019											
The Centre Party	-	-	82	7.23	47	6.45	39	7.25	29	6.67	9.4
The Finns Party	-	-	104	9.17	65	8.92	48	8.92	38	8.74	11.95
The National Coalition Party	-	-	183	16.14	110	15.09	85	15.80	74	17.01	11.62
The Social Democratic Party	-	-	187	16.5	107	14.68	72	13.38	59	13.56	12.12
The Green League	-	-	221	19.5	159	21.81	109	20.26	80	18.39	7.85
The Left Alliance	-	-	110	9.7	84	11.52	70	13.01	58	13.33	5.58
Other	-	-	124	10.93	86	11.80	61	11.34	44	10.11	9.81
None	-	-	123	10.85	71	9.74	54	10.04	46	10.57	31.67

*Statistics Finland 2020, Statistics Finland. (2020). 'Statistics Finland's Databases : Statfin'. https://statfin.stat.fi/PxWeb/pxweb/fi/StatFi

Appendix S2. Weights

Table S2 shows the weights used in our statistical models. We have accounted for the skewness of the data and attrition by using post-stratification weights calibrated on age, gender and education. We first counted the weights by age and gender, and then further multiplied them with the weights for educational level (Deville and Sarndal, 1992).

Table S2. Weights used to calibrate the data

Weights by gender and age group

	male				female			
age	N	%	Population % *	weight	N	%	Population % *	weight
18-24	12	0,028	0,058	2,093	19	0,044	0,055	1,254
25-34	33	0,077	0,092	1,205	45	0,104	0,087	0,832
35-44	37	0,086	0,089	1,038	24	0,056	0,084	1,510
45-54	55	0,128	0,089	0,700	33	0,077	0,088	1,144
55-64	51	0,118	0,091	0,772	43	0,100	0,094	0,944
65-74	46	0,107	0,082	0,764	33	0,077	0,090	1,178
Total	234	0,543	0,502		197	0,457	0,492	

Weights by education level

Education	N	%	Population % *	weight
Primary	27	0,063	0,176	2,79
Upper secondary	118	0,276	0,473	1,72
Higher level	283	0,661	0,35	0,53
Total	428			

*Statistics Finland 2020, Statistics Finland. (2020). ‘Statistics Finland’s Databases: Statfin’. https://statfin.stat.fi/PxWeb/pxweb/fi/StatFin/

Appendix S3. Contextual description

Table S3 presents a summary of some of the key factors related to the adverse events happening during our data collection period, as well as the level of emphasis placed on the environment and climate change in Eurobarometer surveys conducted around the same time as our observation periods. In addition, the inflation and consumer confidence trends are presented in the Figure S1.

Table S3. Contextual description

**Observation period**	**Environment / climate change** (European Commission 2017; 2019; 2020; 2022; 2023)	**COVID-19** (The Finnish Government, 2023; Finnish Institute for Health and Welfare, 2024)	**War** (The Finnish Government, 2023)	**Inflation** (Statistics Finland, 2024a)	**Consumer confidence** (Statistics Finland, 2024b)
**T1 (12/2017)**	14 % consider "the environment, climate and energy issues" as top two issue facing Finland			Moderate inflation	Higher than average (6,8)
**T2 (3/2019)**	35 % consider "the environment, climate and energy issues" as top two issue facing Finland, hopeful times, key topic in the parliamentary elections, and salient in the media			Moderate inflation	Slightly lower than average (-2,3)
**T3 (5/2020)**	24 % consider "the environment and climate change" as top two issue facing Finland	First wave of COVID-19 with low number of cases, relatively high concern (mean 4,0/10), no vaccinations		Moderate inflation	Very low for the past few months (-13,9: -9)
**T4 (12/2021)**	22 % consider "environment and climate change" as top two issue facing Finland	A lot of COVID-19 cases, relatively high concern (mean 4,1/10), majority of Finns vaccinated		Accelerated inflation	Decreased back to lower than average (-3,5)
**T5 (12/2022)**	15 % consider "environment and climate change" as top two issue facing Finland	Low concern (mean 2,4/10)	Concerned about the war impact on: 60% the Finnish economy; 25% national security; 44% European security	High inflation	All time low (-18,5)

Figure S1. Consumer price index* (orange line, 2015=0%) and consumer confidence** (blue line, balance figure)

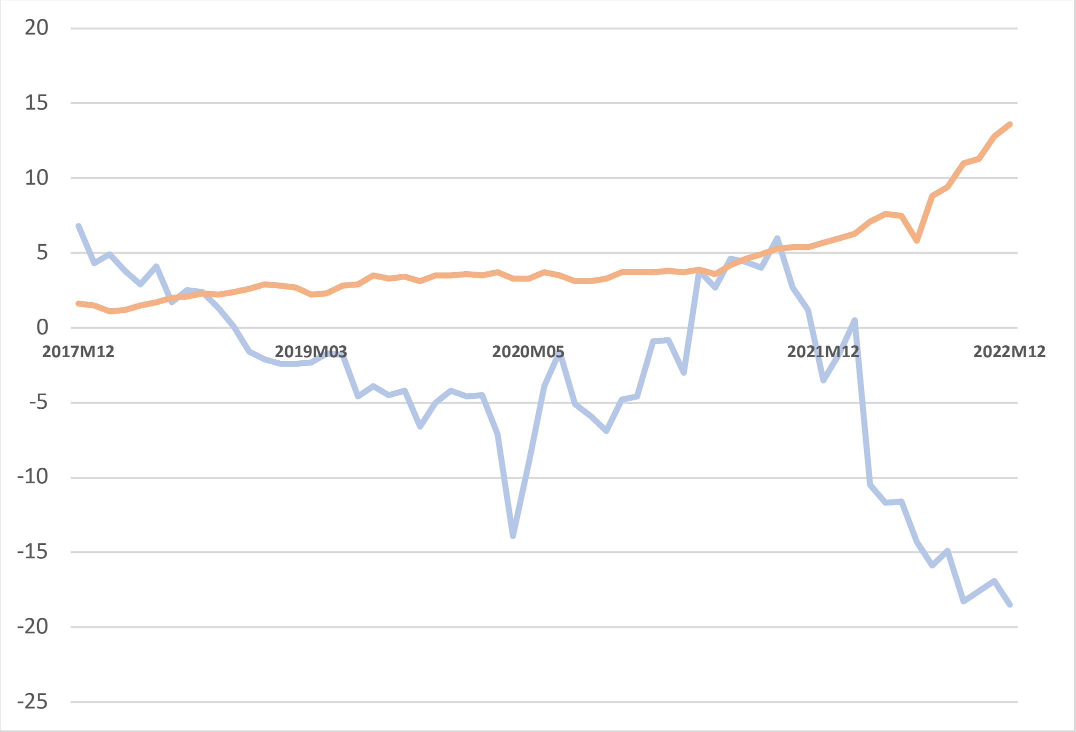

* Statistics Finland, 2024a; ** Statistics Finland, 2024b

Sources

European Commission. (2017). Standard Eurobarometer 88 - Autumn 2017. European Commission.

European Commission. (2019). Special Eurobarometer 486: Europeans in 2019. European Commission.

European Commission. (2020). Standard Eurobarometer 93 - Summer 2020. European Commission.

European Commission. (2022). Standard Eurobarometer 96 – Winter 2021-2022. European Commission.

European Commission. (2023). Standard Eurobarometer 98 – Winter 2022-2023. European Commission.

Finnish Institute for Health and Welfare. (2024). COVID-19 cases in the infectious diseases registry.

Finnish Government. (2023). Citizen Pulse – Round 46 (in Finnish). Helsinki: Finnish Government. https://www.stat.fi/tup/htpalvelut/tutkimukset/kansalaispulssi.html

Statistics Finland. (2024a). Consumer Price Index. https://pxdata.stat.fi/PxWeb/pxweb/en/StatFin/StatFin__khi/statfin_khi_pxt_11xb.px/

Statistics Finland. (2024b). Consumer Chttps://pxdata.stat.fi/PxWeb/pxweb/en/StatFin/StatFin__kbar/statfin_kbar_pxt_11cc.px/

Appendix S4. Robustness checks

Different components of political trust

Figure S2. Economy-over-environment policy preferences by trust in the parliament and round 2017–2023. The predicted scores from the weighted REWB model (M6). Estimates with 95 % confidence intervals.

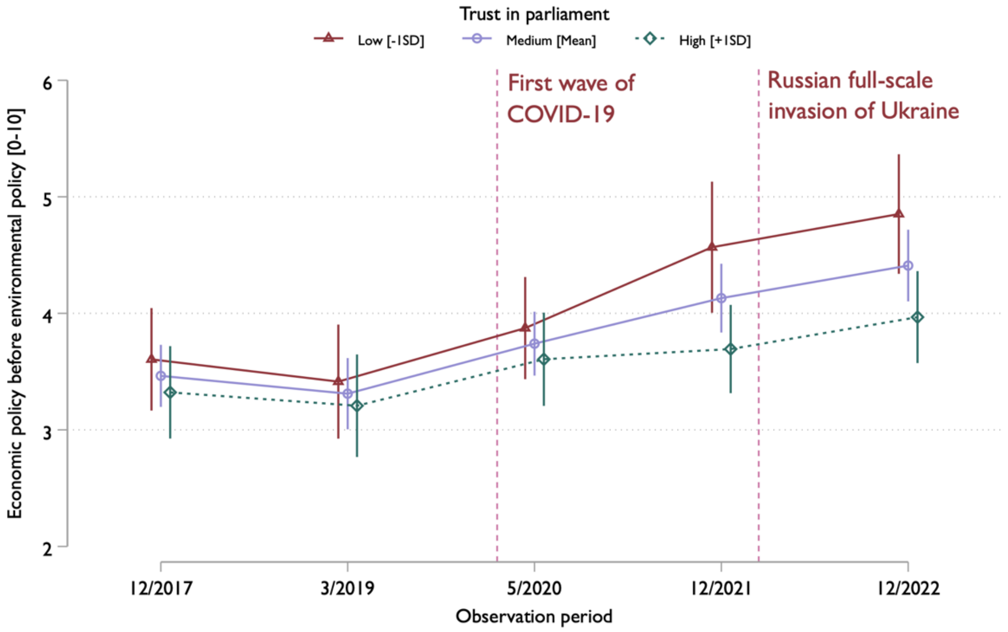

Figure S3. Economy-over-environment policy preferences by trust in politicians and round 2017–2023. The predicted scores from the weighted REWB model (M6). Estimates with 95 % confidence intervals.

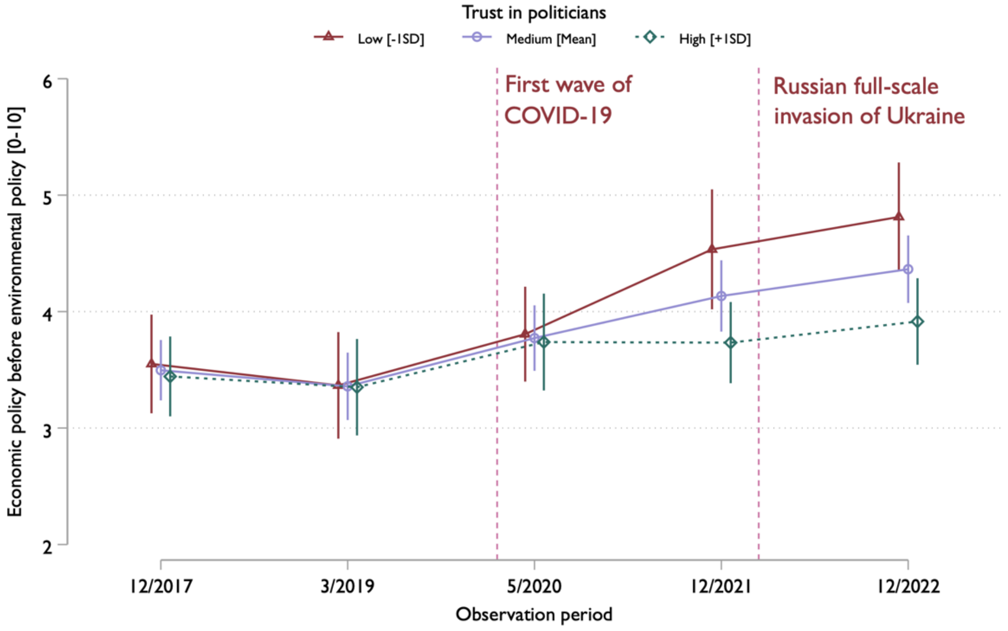

Figure S4. Economy-over-environment policy preferences by trust in political parties and round 2017–2023. The predicted scores from the weighted REWB model (M6). Estimates with 95 % confidence intervals.

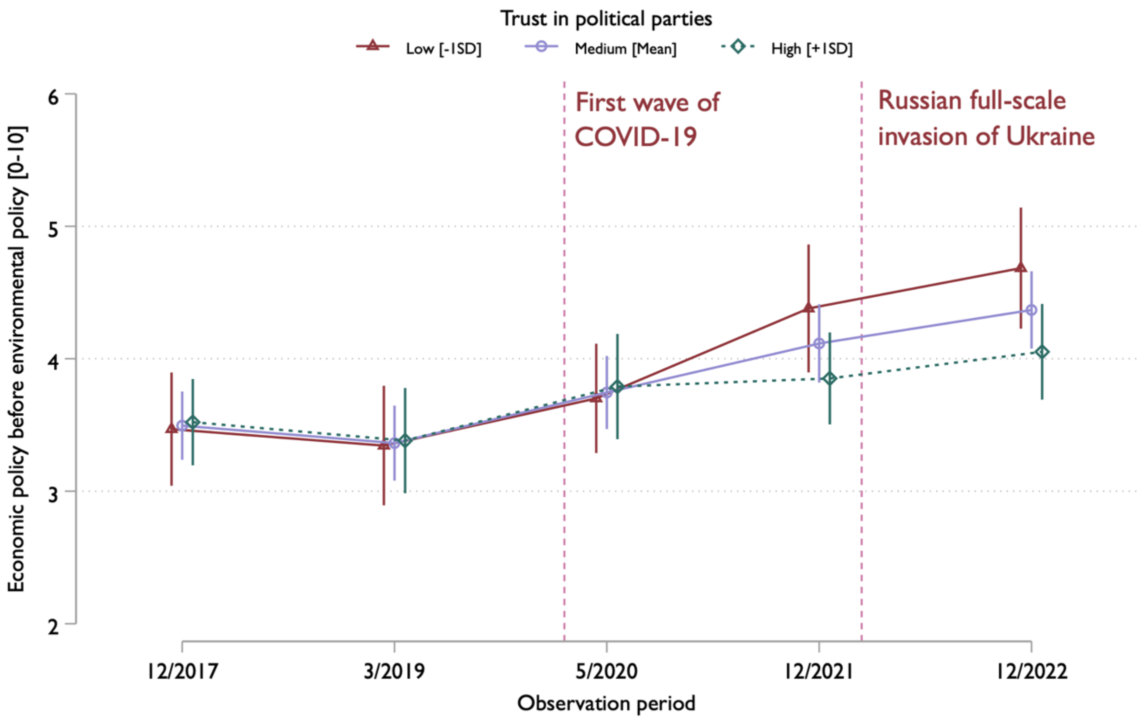

Pre-election political trust

Figure S5. Economy-over-environment policy preferences by political trust at T1 and round 2017–2023. The predicted scores from the weighted REWB model (M6). Estimates with 95 % confidence intervals.

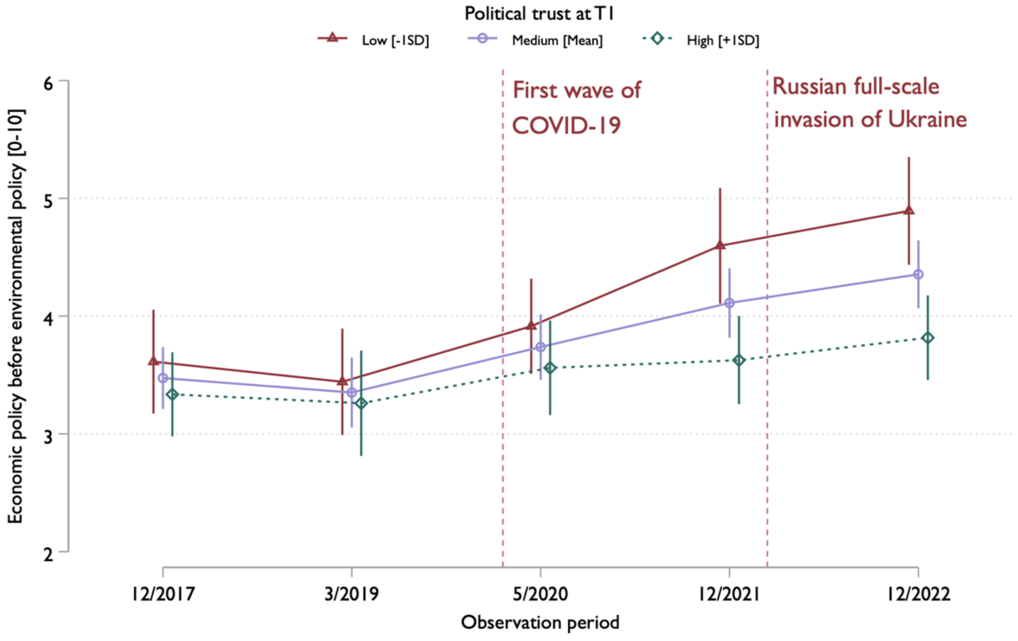

Figure S6. Economy-over-environment policy preferences by political trust at T2 and round 2017–2023. The predicted scores from the weighted REWB model (M6). Estimates with 95 % confidence intervals.

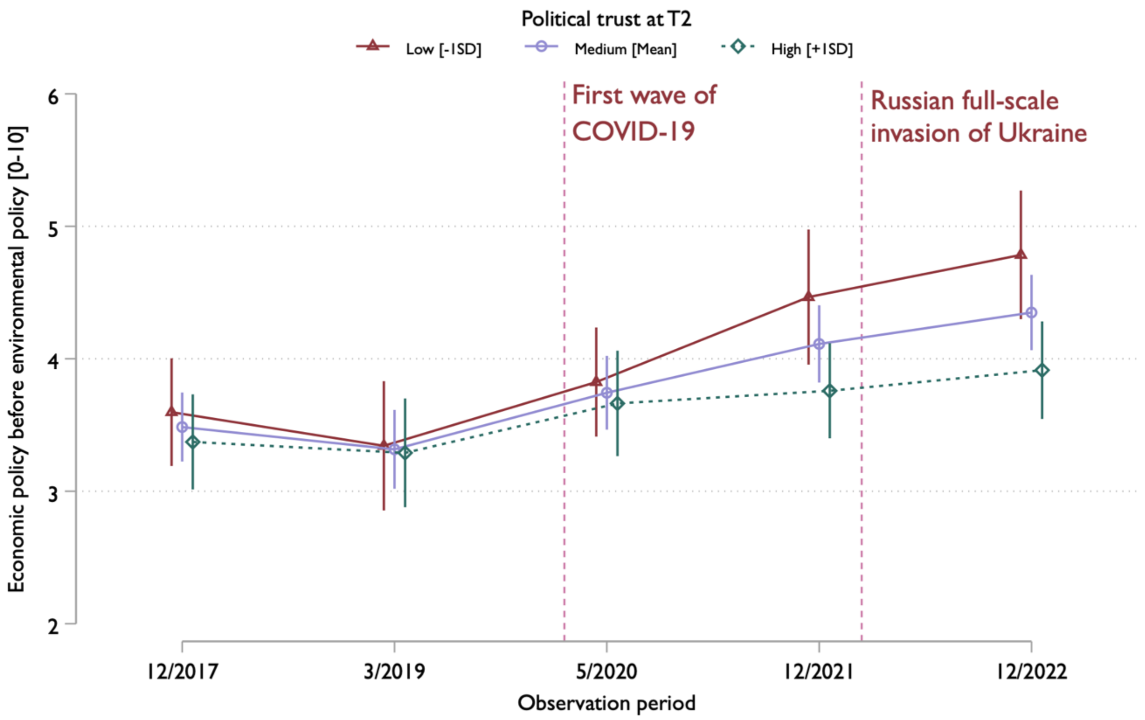

Supplement: Supplementary file 1 — Supplementary file1 (PDF 903 KB) [file 13280_2024_2054_MOESM1_ESM.pdf]
